# Synchronous Multiple Lung Adenocarcinomas: Estrogen Concentration in Peripheral Lung

**DOI:** 10.1371/journal.pone.0160910

**Published:** 2016-08-15

**Authors:** Koei Ikeda, Kenji Shiraishi, Ayaka Yoshida, Yusuke Shinchi, Mune Sanada, Yamato Motooka, Kosuke Fujino, Takeshi Mori, Makoto Suzuki

**Affiliations:** Departments of Thoracic Surgery, Graduate School of Medical Science, Kumamoto University, 1-1-1 Honjo, Kumamoto 860-8556, Japan; Peking University People's Hospital, CHINA

## Abstract

**Background:**

The detection rate of synchronous multiple lung adenocarcinomas (SMLA), which display multiple ground glass opacity nodules in the peripheral lung, is increasing due to advances in high resolution computed tomography. The backgrounds of multicentric development of adenocarcinoma are unknown. In this study, we quantitated estrogen concentration in the peripheral lungs of postmenopausal female patients with SMLA.

**Methods:**

The tissue concentration of estrogens (estrone [E1] and estdadiol [E2]) in the noncancerous peripheral lung were measured with liquid chromatography/electrospray tandem mass spectrometry in postmenopausal female patients with lung adenocarcinoma. The expression levels of *CYP19A1* in the normal lung were also quantitated with real-time PCR. Thirty patients with SMLA and 79 cases of control patients with single lung adenocarcinoma were analyzed.

**Results:**

The concentrations of E1 and E2 in the noncancerous tissue were significantly higher in SMLA cases than control cases (P = 0.004 and P = 0.02, respectively). The minor allele (A) of single nucleotide polymorphism rs3764221 were significantly associated with higher concentration of E1 and E2 (P = 0.002 and P = 0.01, respectively) and higher CYP19A1 mRNA expression (P = 0.03).

**Conclusion:**

The tissue estrogen concentration of peripheral lung was significantly higher in SMLA than control cases. The high concentration of estrogen may be one of the causes of multicentric development of peripheral lung adenocarcinomas.

## Introduction

Recent advances in high resolution computed tomography (HRCT) and computed tomography (CT) screening for lung cancer have led to an increased detection rate of synchronous multiple lung adenocarcinomas (SMLA). SMLAs often display multiple ground-glass opacity (GGO) nodules in the peripheral lung and are diagnosed as adenocarcinoma (AD) with a lepidic pattern or atypical adenomatous hyperplasia (AAH) [1,2]. These tumors are found more frequently in women and tend to harbor EGFR mutation compared with other types of lung cancer [3]. However, the background for multicentric development of peripheral lung neoplastic lesion is unknown.

For women, lung cancer is the fifth leading-site in cancer incidence and is continuously increasing in Japan. Especially, adenocarcinoma is increasing in women [4]. The epidemiology is different in women, with a relatively low influence from tobacco smoking. Many studies have shown that estrogen played an important role in lung cancer carcinogenesis [5,6]. Polymorphisms in genes involved in estrogen metabolism are thought to be associated with circulating estrogen levels. Aromatase (*CYP19A1*) is a cytochtome P-450 enzyme that converts androstendione and testosterone to estrone and estradiol. We previously reported a significant association between SMLA risk and a single nucleotide polymorphisms (SNP) rs3764221, in *CYP19A1* [7]. The use of liquid chromatography/electrospray tandem mass spectrometry (LC-MS/MS) allowed us to measure tissue estrogen concentration, which is too low to measure using immunological methods. In this study, we quantitated estrogen concentrations using LC-MS/MS in non-cancerous peripheral lung tissue of postmenopausal *w*omen with SMLA and compared with control postmenopausal female patients with single tumor lung AD to determine an association of estrogen concentration and multicentric development of lung ADs. We conducted this analysis limited to postmenopausal women because (1) SMLA is frequently diagnosed in postmenopausal women [3,7], (2) the epidemiology of lung cancer in men is greatly affected by tobacco smoking, (3) the concentration of estrogen in lung tissue was reported to be significantly higher in men than in postmenopausal women, which is because serum androgen concentration, known to be major substrate of local estrogen production, is significantly higher [8].

## Material and Methods

### Study subjects

A case was classified as SMLA if two or more GGO nodules in peripheral lung were histologically diagnosed as AD with lepidic feature. The criteria of Martini and Melamed were used to confirm that the multiple lesions were not metastatic [9]. To avoid the influence of ovarian estrogen secretion, patients younger than 55 years old were excluded from this study. The patient who received preoperative chemotherapy or oral or inhaled steroid therapy were also excluded. Thirty SMLA cases were selected from the consecutive patients who underwent surgical resection in the Department of Thoracic Surgery of Kumamoto University Hospital from 2012 to 2015. Among these 30 patients, two GGO nodules were clinically diagnosed as lung ADs in 15 cases, three of more nodules were recognized in15. In five cases (16.7%), AAH was detected within the resected specimens. The pathological diagnosis of representative nodules (the most invasive or largest among the resected nodules), in accordance with the new classification system recently proposed by Travis et al. [10] were AD in situ, non-mucinous in 8 cases, minimally invasive AD, non-mucinous in 6 cases, invasive AD, in 16 cases. The control group comprised 79 postmenopausal female patients with single-tumor lung AD who received curative surgical resection during 2013 to 2015. Their subtypes of adenocarcinoma were AD in situ, non-mucinous in 14 cases, minimally invasive AD, non-mucinous in 6 cases and invasive AD, in 59 cases. All of the analyzed patients were Japanese. The study protocol for genetic analysis of the blood samples and resected specimens was approved by the Ethics Committee of Kumamoto University Hospital in May 2011. All patients provided written informed consent for their data and materials to be used for research purpose.

### Measurement of Estrogen concentration

Non-cancerous peripheral lung tissue was harvested in 5mm square sections and frozen immediately in liquid nitrogen. Tissue concentrations of E1 and E2 were measured by LC–MS/MS analysis, in ASKA Pharma Medical (Kawasaki, Japan), as described previously [8, 11]. Briefly, the frozen tissue was homogenized in 1 mL of 0.1M KH2PO4 solution by Ultra-Turrax homogenizer. The steroids were extracted by 4 mL of methyl tert-butyl ether (MTBE) from the suspension and eluted with 1 mL of methanol/distilled water/pyridine mixture (90:10:1, v/v/v). The sample was allowed to react with 50 μL of pentafluoropyridine, 40 μL 1M sodium hydroxide solution, 100 μL acetonitrile and 20 μL ethanol for 20 min at room temperature. The residue was dissolved in 0.5 mL distilled water and allowed to react with 0.14 mL of the reaction reagent mixture [1 mg of 2-methyl-1-hydradino-pyridine in 2.6 mL acetonitrile and 0.2 mL of acetonitrile/trifluoro acetic acid mixture (99:1, v/v)] for 1 h at room temperature. The residue was applied to an Oasis WCX cartridge and was derivatized E1 [E1-3-tetrafluoropyridil-17-(1’-methylpyridinium-2’)-hydrazone and dissolved in 0.1 mL acetonitrile/ distilled water mixture (7:3, v/v) and 20 μL of the solution was subjected to an LC-MS/MS. The derivatized E2 fraction was allowed to react with 50 μL of the reaction reagent mixture (80 mg of 2-methyl-6-nitrobenzoic anhydride, 20 mg of 4-dimethylaminopyridine, and 40 mg of fusaric acid in 1 ml of acetonitrile) and 10 μl of triethylamine for 30 min. at room temperature. After the reaction. The cartridge was eluted in 2.5 mL of ethyl acetate /hexane mixture (9:11, v/v). The residue was dissolved in 0.1 mL acetonitrile/ distilled water mixtuer (4:1, v/v) and 20 μL of the solution was subjected to an LC-MS/MS. An API-5000 triple stage quadrupole mass spectrometer (SCIEX, Framingham, MA, USA) equipped with a positive ESI source and a Shimadzu HPLC system (SCL-10Avp system controller, LC-20AD pump, SIL-HTc column oven, CTO-20A auto-sampler, Shimazu GLC, Tokyo, Japan) was used. A Kinetex C18 column (2.6 μm, 150×3 μmm. Shimazu) was used at 50°C. The lower limit of quantitation of E1 and E2 was 0.05 pg/tube and 0.03 pg/tube, respectively.

### Genomic analysis

Genomic DNA was extracted from whole blood using QiaAmp DNA Blood Mini Kit (QIAGEN, Tokyo, Japan) according to the supplier’s instructions. SNPs were identified by using a 5’ nuclease assay with a TaqMan MGB probe. The reactions were performed in an ABI 7500HT (Applied Biosystems, Foster City, CA, USA). The SNP primers and TaqMan MGB probes were provided by the Assay on-Demand service (Applied Biosystems). The 5’ nuclease assays were performed with 10ng genomic DNA, 1x Taqman Genotyping Master Mix (Applied Biosystems), and 1x primer/probe mix according to the manufacturer’s instructions.

### CYP19A1 expression analysis

To confirm the effects of the SNP in the *CYP19A1*, the expression levels of *CYP19A1* gene in noncancerous lung were quantified by real-time RT-PCR. The FastPure RNA Kit (Takara Bio, Shiga, Japan) was used to isolate total RNA from the frozen tissue. Levels of *CYP19A1* mRNA were measured by real-time RT-PCR. Total RNA (1μg) was reversed transcribed using the SuperScript III First-Strand System (Life Technologies Inc., Rockville, MD, USA). Quantitative RT-PCR reactions were performed on the ABI ViiA^™^ 7 instrument using TaqMan^®^ Universal Master Mix and gene-specific primer mixes (both from ABI): *CY19A1* (Hs00903413_m1), The Ct values for each gene were normalized to the housekeeping gene *GAPDH* (Hs02758991_g1), and the fold change in the transcript level was caluculated using the ΔΔCt method. Expression levels were calculated relative to those from the lung AD cell line H358.

### Statistical analysis

Data are expressed as median (minimum–maximum) value for continuous data and as numbers and percentages for categorical data. All statistical analyses were performed by using the SPSS program for Windows (version 18 statistical software; Texas Instruments, IL, USA). The differences of estrogen concentrations and relative expression of *CYP19A1* mRNA were evaluated by using the Mann-Whitney test, and categorical data were compared using the chi-square test.

## Results

### Clinical characteristics

The characteristics of all patients in the cases and the control groups are shown in Table 1. There were no significant differences between the groups with regard to age, smoking status or a familial history of lung cancer. *EGFR* mutations were detected in at least one nodule in 17 of the 30 cases of SMLA (56.7%), which was not significantly different to that of Control cases (59.5%). Heterozygous and homozygous carriers of a minor allele (A) of SNP rs3764221 of *CYP19A1* were significantly frequent among patients with multiple ADs than control group, but the difference was not significant (P = 0.03).

**Table 1:** Comparison of clinical characteristics of synchronous multiple lung adenocarcinomas (SMLA) versus Control (single-tumor lung adenocarcinoma cases).

Variables		SMLA	Control	P value
**Age(mean±SD)**		71.1±5.6	71.3±7.1	N.S.
**Smoking history**	negative	27 (90)	78 (98.7)	
	positive	3 (10)	1 (1.3)	N.S.
**Pathological stage**	IA	29	62	
	IB	1	9	
	IIA	0	3	
	IIB	0	1	
	IIIA	0	4	N.S.
**EGFR mutation**	negative	13 (43.3)	32 (40.5)	
	positive	17 (56.7)	47 (59.5)	N.S.
**Familial history of lung cancer**	negative	24 (80)	68 (86.1)	
	positive	6 (20)	11 (13.9)	N.S.
**SNP rs3764221 of *CYP19A1***	AA/AG	20 (66.7)	33 (41.8)	
	GG	10 (33.3)	46 (58.2)	0.03

### Estrogen concentrations in noncancerous lung

**Fig 1:**
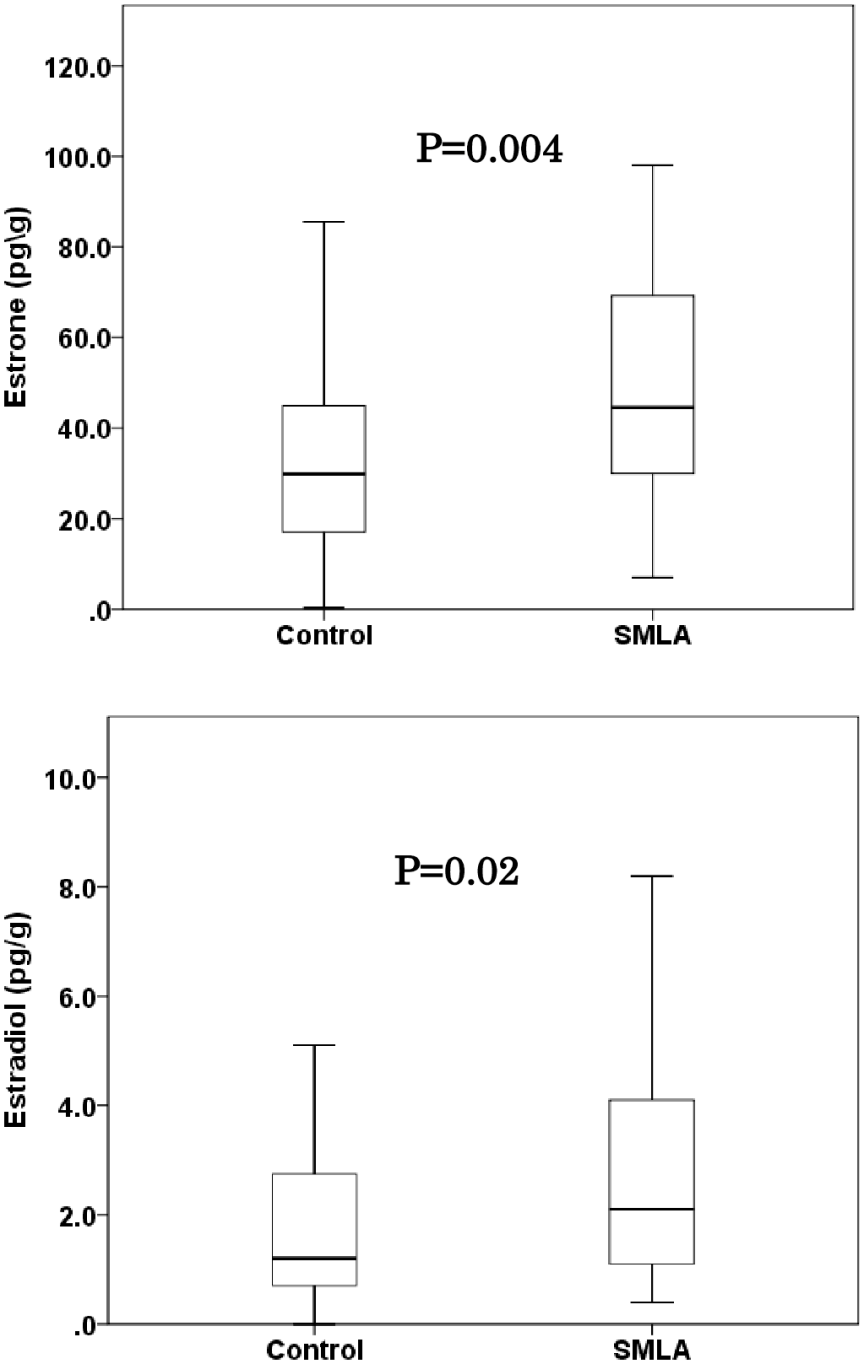
Tissue concentration (pg/g) of Estrone and Estradiol in the noncancerous peripheral lung of SMLA cases and control cases using LC–MS/MS analysis. Data are represented as box and whisker plots. The median value was represented by a horizontal line in the box pot, and gray box denoted the 75th (upper margin) and 25th percentiles of the values (lower margin), respectively. The upper and lower bars indicated the 90th and 10th percentiles, respectively. The statistical analysis was performed using a Mann-Whitney test. P-value less than 0.05 was considered significant.

**Fig 2:**
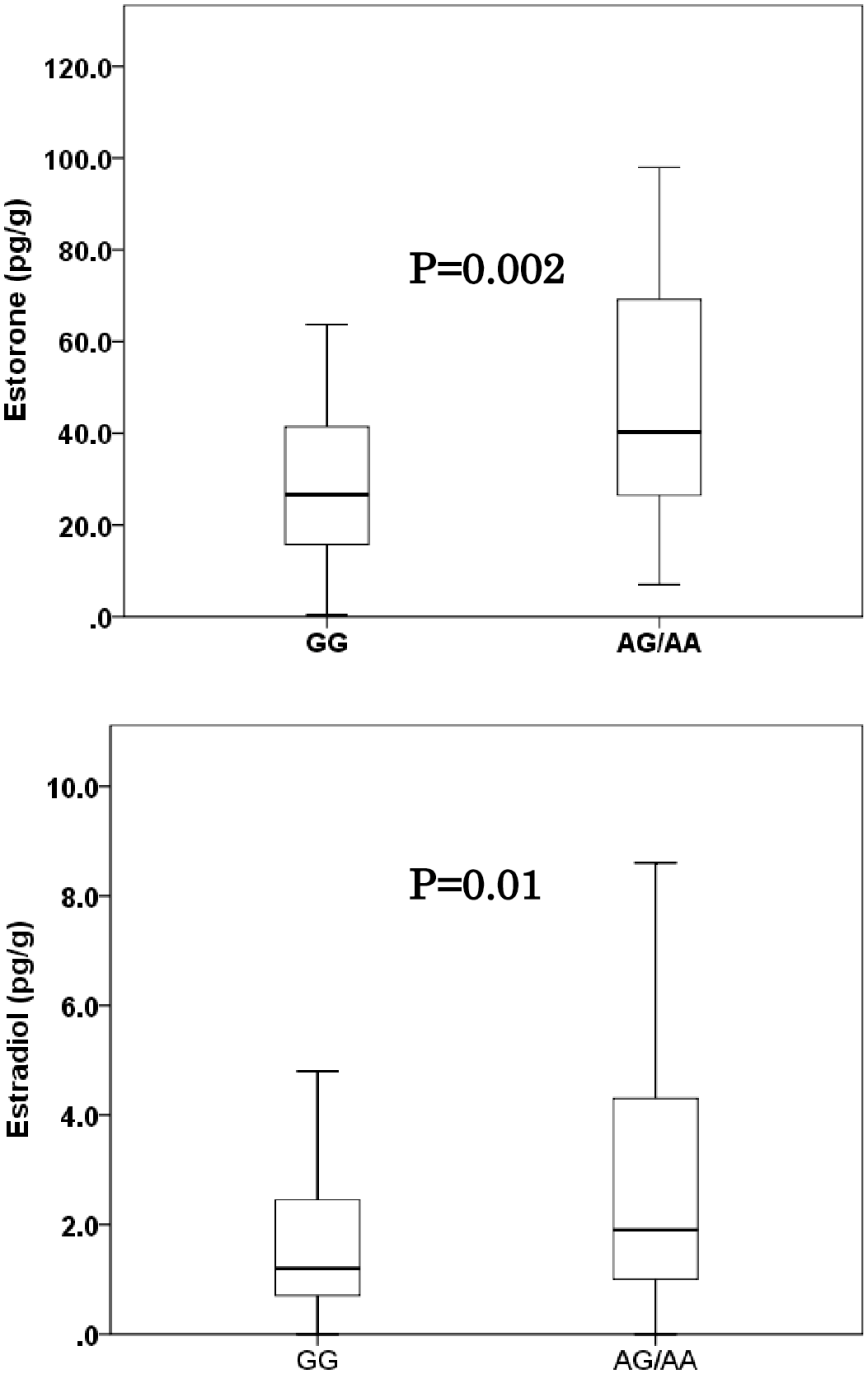
Tissue concentration (pg/g) of Estrone and Estradiol in the noncancerous peripheral lung, according to the minor allele (adenine) of a SNP rs3764221 of *CYP19A1* using LC–MS/MS analysis. Data are represented as box and whisker plots. Data are represented as box and whisker plots. The median value was represented by a horizontal line in the box pot, and gray box denoted the 75th (upper margin) and 25th percentiles of the values (lower margin), respectively. The upper and lower bars indicated the 90th and 10th percentiles, respectively. The statistical analysis was performed using a Mann-Whitney test.

The levels of E1 and E2 in each cases showed a linear relationship (r = 0.839, p<0.001). The distributions of tissue estrogen concentration in the noncancerous peripheral lung of patients of SMLA cases and the control patients are shown in Fig 1. The median concentration (minimum–maximum) of E1 and E2 in the noncancerous lung of SMLA cases were 45.2 (7.0–216.9) pg/g and 2.2 (0.4–8.6) pg/g, respectively. The concentration of E1 and E2 in the noncancerous lung of control cases were 29.0 (0.4–183.8) pg/g and 1.2 (0.0–9.5) pg/g, respectively. The concentration of E1 and E2 were significantly higher in SMLA cases than that of control cases (P = 0.004 and P = 0.02, respectively). The estrogen concentrations were not significantly associated with age, smoking status, pathological stage, familial history of lung cancer or *EGFR* mutation status. (S1 Table) The distributions of tissue estrogen concentration of all analyzed lung adenocarcinoma patients according to the allele type of rs3764221 of *CYP19A1* gene is shown in Fig 1. The median concentration (minimum–maximum) of E1 and E2 in the noncancerous lung of homozygous carriers of major allele of SNP rs3764221(GG) was 26.3 (0.3–139.5) and 1.2 (0.0–6.7), respectively. That of heterozygous and homozygous carriers of minor allele (AA/AG) were and 40.1 (7.0–216.9) and 1.8 (0.0–9.5), respectively. The minor allele of SNP rs3764221 (A) were significantly associated with higher concentration of E1 and E2 (Fig 2: P = .002 and P = 0.01, respectively).

### *CYP19A1* mRNA Expression in noncancerous Lung

**Fig 3:**
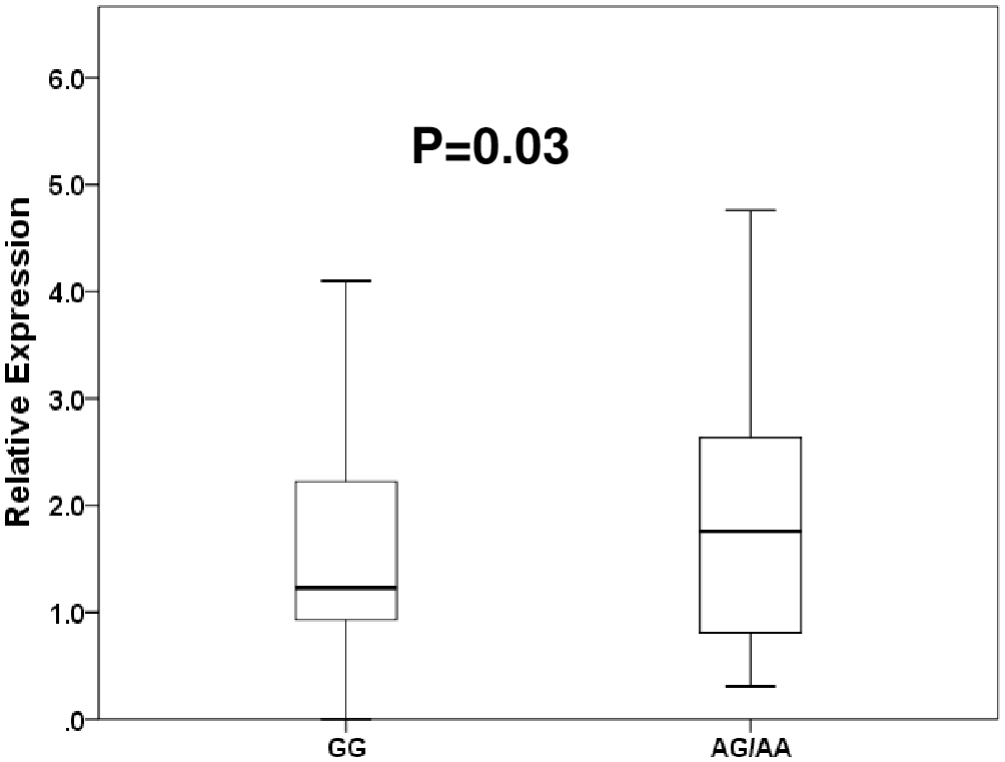
Distribution of relative expression of *CYP19A1* messenger RNA using real-time PCR in noncancerous tissues of lung cancer patients divided by the major allele (guanine) versus homozygosity and heterozygosity of the minor allele (adenine) of a rs3764221 in *CYP19A1*. Data are represented as box and whisker plots. The median value was represented by a horizontal line in the box pot, and gray box denoted the 75th (upper margin) and 25th percentiles of the values (lower margin), respectively. The upper and lower bars indicated the 90th and 10th percentiles, respectively. The statistical analysis was performed using a Mann-Whitney test.

The distributions of *CYP19A1* expression in the noncancerous tissue of patients with lung cancer are shown in Fig 3. The median value (minimum–maximum) of relative quantities of *CYP19A1* expression in homozygous carriers of major allele of SNP rs3764221(GG) and heterozygous and homozygous carriers of minor allele (AA/AG) were 1.2(0–5.2) and 1.8 (0–9.1), respectively. The minor allele of SNP rs3764221 (adenine) was significantly associated with higher expression of *CYP19A1* mRNA (P = 0.03).

## Discussion

Few study have described the genetic or environmental background of SMLA. The present study suggests that excessive estrogen is one cause of multicentric development of lepidic type lung AD or AAH in the peripheral lung. Our previous report showed that the minor allele of SNP rs3764221 in the *CYP19A1* gene is significantly associated with SMLA risk [7]. In the present study, we focused on estrogen concentration of the background lung of AD, and could demonstrate the association between estrogen concentration in the peripheral lung and tumor multiplicity in the postmenopausal female patients of lung AD.

Lung cancer in women have several unique characteristics compared with in men. Never smoker is more frequent in female lung cancer patients and AD with lepidic growth, which frequently have EGFR mutation, is more popular histology in women [12,13]. The influences of estrogen on the etiology of lung cancer is conflicting. In mouse model, estrogen promotes tumorigenesis of lung ADs [14]. In the population-based Geneva Cancer Registry, compared with expected outcomes in the general population, breast cancer patients receiving antiestrogen treatment for breast cancer had lower lung cancer mortality [15]. In the Women's Health Initiative trial, treatment with estrogen plus progestin in postmenopausal women increased the number of deaths from lung cancer [16]. On the other hand, a large population-based case-control study indicated that women who continue to produce estrogen have a lower lung cancer risk [17]. In the prospective California Teachers Study cohort, menopausal hormone therapy decreased lung cancer mortality [18].

In postmenopausal women, estrogen is mainly synthesized by aromatase in non-gonadal tissue such as adipose tissue, adrenal grand and lung. *CYP19A1* has multiple promoters, and these promoters are expressed in a tissue specific manner [19]. We have shown that a SNP rs3764221, which is in the I4 promoter in intron1 of CYP19A1, was associated with SMLA risk, and hypothesized that this SNP is specifically associated with local elevation in *CYP19A1* expression in peripheral lung. In this study, the minor allele (A) of rs3764221 was associated with locally elevated *CYP19A1* mRNA and higher estrogen concentration in peripheral lung. The excessive localized production of estrogen might cause multicentric development of GGO lesion in the context of field cancerization. If local elevations in estrogen concentration are associated with tumorigenesis and disease progression of SMLA, then estrogen and aromatase became possible targets for prevention and treatment. Indeed, several studies have reported effectiveness of ER blockers or aromatase inhibitors for lung cancer treatment [20–23]. For the cases of SMLA in which surgical complete resection is impossible because of too many nodules in multiple lobes, such drugs might effective for disease control.

We recognize certain limitations of our study. The greatest one is that, the impact of estrogen concentration for the risk of SMLA could not assessed because the lung samples from normal noncancerous patients were not available. In this study, we could only compare between SMLA and single tumor lung AD patients to assess the associations between estrogen concentration and tumor multiplicity.

In conclusion, the concentration of estrogen in the peripheral lung tissue was higher in SMLA cases than in single-tumor lung AD cases in the limited analysis of postmenopausal women. SNP rs3764221, which is located in the *CYP19A1* gene, was significantly correlated with higher expression of *CYP19A1* mRNA and higher concentration of estrogens in lung tissue. Polymorphisms of the *CYP19A1* gene could lead to locally elevated estrogen levels in the peripheral lung, and this localized overproduction of estrogen may be one of the factors associated with multicentric development of ADs in peripheral lung.

## Supporting Information

S1 Table individual data of analyzed SMLA cases and control. (CSV)
